# The Importance of the Tumor Microenvironment to Understand Tumor Origin, Evolution, and Treatment Response

**DOI:** 10.3390/cancers14081983

**Published:** 2022-04-14

**Authors:** Jose M. Ayuso, Ignacio Ochoa Garrido

**Affiliations:** 1 Department of Dermatology, University of Wisconsin-Madison, Madison, WI 53705, USA; 2 Department of Pathology and Laboratory Medicine, University of Wisconsin-Madison, Madison, WI 53705, USA; 3 Carbone Cancer Center, University of Wisconsin-Madison, Madison, WI 53705, USA; 4 Tissue Microenvironment (TME) Group, Aragon Institute of Engineering Research (I3A), University of Zaragoza, 50018 Zaragoza, Spain; 5 Institute for Health Research Aragón (IIS), 50018 Zaragoza, Spain; 6 Centro de Investigacion Biomedica en Red en Bioingenieria, Biomateriales y Nanomedicina (CIBER-BBN), 50018 Zaragoza, Spain

During the second half of the twentieth century, oncology adopted a tumor-centric approach to cancer treatment, focusing primarily on the tumor cell to identify new therapeutic targets [1]. However, since the 2000s, we have seen a gradual shift in this paradigm with numerous studies highlighting the importance of the tumor microenvironment in tumor progression, patient prognosis, and therapy response [2,3]. Solid tumors are highly complex systems where numerous cell types and microenvironmental factors are intertwined, potentially affecting tumor evolution, treatment response, and patient outcome [4]. Stromal cells such as fibroblasts and immune cells can stimulate or suppress tumor growth and are currently being used as therapeutic targets in numerous studies and clinical trials [5].

The emergence of new technologies to spatially resolve and interrogate the tumor microenvironment has provided researchers a new toolbox to evaluate the TME in an unapparelled way [6,7,8]. Spatial transcriptomics techniques allow the user to evaluate gene or protein expression in multiple tissue compartments (e.g., tumor core, vasculature, surrounding stroma, immune population), potentially providing the user with clinically-relevant information. In this context, Monkman et al. used spatial transcriptomics to evaluate gene expression in a small patient cohort diagnosed with Non-Small-Cell Lung Cancer. Their spatial transcriptomics analysis revealed the capacity of some tumors to exclude immune effector cells (e.g., CD8) from the tumor mass. They used this information to develop a transcriptomic signature based on the expression of CD3 and ICOS to predict patient overall survival [9]. Advances in microtechnologies and in vitro culture have led to the development of advanced in vitro tools to capture the tumor microenvironment and study the role of stromal and microenvironmental factors in tumor evolution [10]. Sadangi et al. leveraged the use of microfluidic platforms to illustrate the role of dermal fibroblasts and keratinocytes in melanomagenesis [11]. BRAF mutations are found in more than 90% of melanomas, however these mutations are also present in other benign lesions such as moles. After BRAF mutation, melanocytes exhibit a short burst of cell proliferation that eventually leads to cellular senescence and growth arrest. This process is known as oncogene-induced senescence (OIS) and is considered a protective mechanism that prevents BRAF-mutated melanocytes from evolving into invasive melanoma. However, the mechanisms that allow some BRAF-mutated melanocytes to escape OIS and lead to melanoma are poorly understood. Sadangi et al. demonstrated that keratinocytes and dermal fibroblasts reduce/prevent senescence in BRAF-transduced melanocytes. Although the authors did not identify the specific molecular driver controlling this process, they identified multiple genetic changes caused by the presence of keratinocytes, including downregulation of P16, calreticulin, and several CDKs [11]. Similar to stromal cells, local biochemical factors such as oxygen, pH, and nutrients also modulate tumor evolution. In this context, oxygen concentration and local hypoxia regulate tumor response to radiotherapy, which relies on the use of electromagnetic radiation to cause DNA damage by direct and indirect mechanisms, eventually leading to cell death. Grenz-ray therapy (GT) uses low-energy radiation for multiple applications in dermatology including allergic diseases, such as eczema, contact dermatitis, psoriasis, and lentigo maligna/lentigo maligna melanoma. GT offers higher linear energy transfer and relative biological effectiveness compared with other radiotherapy techniques such as X-rays. However, selecting the optimal radiation dose to selectively destroying the tumor cells while minimizing radiation exposure to normal tissue can be challenging since local hypoxia within the tumor reduces the efficacy of radiotherapy. Thus, Chan et al. developed a methodology to calculate the GT dose required to damage and destroy cancer cells depending on the oxygen concertation [12]. They combined experimental observations with Monte Carlo simulations across multiple cell sources to evaluate oxygen-dependence during GT. Their results demonstrated that oxygen-dependence decreases as radiation energy is decreased, making GT a promising approach for tumors with lower oxygen concentration. Son et al. explored the therapeutic potential of antibodies to increase radiotherapy sensibility [13]. More specifically, they evaluated the use of trastuzumab (i.e., anti-HER2 antibody used to treat HER+ breast cancer) in combination with fractioned X-ray radiotherapy. They demonstrated that pre-treatment with trastuzumab reduced the hypoxic fraction of the tumor as well as tumor viability both in vitro and in vivo.

The tissue microenvironment also plays a critical role in tumor metastasis. Breast cancer commonly metastasizes, leading to a much worse patient prognosis. However, the factors allowing breast cancer cell survival, and, more importantly, outgrowth in the bone microenvironment are poorly understood. Hughes et al. combined in vivo models, in vitro experiments, and mathematical simulation to explore the role of the bone microenvironment in metastatic breast cancer evolution [14]. Using animal models, they demonstrated that metastatic breast cancer cells commonly cluster around osteoblast-rich regions, and osteoblast density positively correlated with metastatic outgrowth. Next, their in vitro results showed that osteoblasts protected metastatic breast cancer cells from oxidative stress through the formation of gap junctions, potentially providing a glutathione reservoir for the cancer cells. Finally, they used mathematical modelling to develop a model to predict the likelihood of developing metastatic growth based on the number of osteoblasts in the surrounding bone microenvironment. Sethakorn et al. reviewed the microenvironment of solid tumors metastasis within the bone marrow. In their article they described the complex crosstalk between stroma and cancer cells and analyze the current models that enable its study. They also illustrate the role of several molecular pathways (such as TGF-β, BMP, WNT, chemokines, etc.) in the bone marrow tumor microenvironment. Understanding these pathways is key for the development of new targeted therapeutical strategies [15].

Glioblastoma is the most common and aggressive brain tumor [16]. Currently available treatments are based on an aggressive combination of brain surgery, radiotherapy, and chemotherapy, that only provides an overall survival of approximately one year after diagnosis [16]. Previous studies have suggested that the surrounding microglia promotes tumor growth, invasion, and treatment resistance, but the specific mechanisms remain poorly understood. Nuñez et al. demonstrated that the presence of tumor-infiltrating microglia led to tumor proliferation and invasion by focal adhesion kinase (FAK) and proline-rich tyrosine kinase 2 (PyK2) signaling [17]. Using RT-qPCR and western blot in human specimens, they demonstrated a positive correlation between FAK/PyK2 activation in tumor cells and platelet-derived growth factor β(PDGFβ), stromal-derived factor 1α (SDF-1α), IL-6, IL-8, and epidermal growth factor (EGF) secretion in microglia. Next, they derived glioblastoma cell lines from human samples and used siRNA and pharmacological inhibitors to demonstrate that EGF and IL-6 regulated FAK/PyK2-dependent glioblastoma cell proliferation, viability and invadopodia formation. Balaziova et al. studied the role of mesenchymal cells in glioblastoma progression and blood-brain barrier disruption. They observed fibroblast activated protein (FAP)-expressing mesenchymal cells around the vasculature, and the presence of FAP+ mesenchymal cells positively correlated with destabilized vasculature and glioblastoma cell invasion. Protein secretion analysis revealed that these mesenchymal cells produced multiple proangiogenic factors including angiopoietin-2, VEGF, or endothelin-1, whereas thee expression of anti-angiogenic factors was downregulated (e.g., endostatin-2, vasohibin, and IGFBP3). Their in vitro experiments demonstrated that FAP+ mesenchymal cell conditioned media induced chemotactically-directed angiogenic sprouting [18].

Animal models provide a versatile platform to evaluate the role of the TME. Advances in genetic engineering and tissue manipulation are leading to advanced models to mimic complex tumor behaviors [8]. Bella et al. reviewed advances in animal modelling to study peritoneal carcinomatosis [19]. In this context, gastrointestinal (e.g., colorectal cancer) and gynecological malignancies (e.g., ovarian cancer) can spread to the peritoneal cavity, which significantly worsens patient prognosis and limits the number of therapeutic options available. In this review, Bella et al. analyzed advances in the field and discuss the advantages and limitations of the animal models available to study peritoneal carcinomatosis. Qiu et al. developed a new animal model to capture the hepatocellular carcinoma (HCC) microenvironment [20]. First, they generated a three-dimensional sheet-like human HCC organoid in vitro, combining luciferase-expressing Huh7 cells, human iPSC-derived endothelial cells (iPSC-EC), and human iPSC-derived mesenchymal cells (iPSC-MC). The authors used a ultra-pure alginate gel as scaffolding material and was implanted in immune-deficient mice. Using the model, the authors demonstrated that iPSC-MC induce HCC growth and tumor invasion. Additionally, they also observed that liver fibrosis promotes HCC tumor growth. The main advantage of this model is the capacity to control the components of the TME, allowing the user to evaluate the role of multiple cell populations in tumor progression.

Overall, this special issue contributes to highlight the critical role of the tumor microenvironment in multiple aspects of tumor biology, from tumorigenesis and tumor growth to cancer metastasis and therapy resistance. As new studies continue to decipher the mechanisms driving these interactions, emerging therapeutic targets should emerge, which in turn could lead to new and more effective therapies that improve patient prognosis.
